# *PPM1D* Mutation as a Distinct Feature of Myeloid Neoplasms in B-Cell Non-Hodgkin Lymphoma Patients: A Retrospective Analysis

**DOI:** 10.3390/cancers17071185

**Published:** 2025-03-31

**Authors:** Heyjin Kim, Jin Kyung Lee, Young Jun Hong, Hye Jin Kang, Byung Hyun Byun, Seung-Sook Lee

**Affiliations:** 1 Department of Laboratory Medicine, Korea Cancer Center Hospital, Korea Institute of Radiological and Medical Sciences, Seoul 01812, Republic of Korea; heyjin@kirams.re.kr (H.K.); clinchem@kirams.re.kr (Y.J.H.); 2 Medical Science Demonstration Center, Korea Cancer Center Hospital, Korea Institute of Radiological and Medical Sciences, Seoul 01812, Republic of Korea; 3 Division of Hematology-Oncology, Department of Internal Medicine, Korea Cancer Center Hospital, Korea Institute of Radiological and Medical Sciences, Seoul 01812, Republic of Korea; hyejin@kirams.re.kr; 4 Department of Nuclear Medicine, Korea Cancer Center Hospital, Korea Institute of Radiological and Medical Sciences, Seoul 01812, Republic of Korea; nmbbh@kirams.re.kr; 5 Department of Pathology, Korea Cancer Center Hospital, Korea Institute of Radiological and Medical Sciences, Seoul 01812, Republic of Korea; sslee@kirams.re.kr

**Keywords:** myeloid neoplasms post cytotoxic therapy, therapy-related myeloid neoplasms, B-cell non-Hodgkin Lymphoma, *PPM1D* mutation, *TP53* mutation

## Abstract

Background/Objectives: Myeloid neoplasms are the most common secondary blood cancer in B-cell non-Hodgkin lymphoma (BNHL) patients treated with cytotoxic therapies. We aimed to characterize the genetic and clinicopathologic features of myeloid neoplasms arising after B-cell non-Hodgkin lymphoma (MN-BNHL) by comparing their features with myeloid neoplasms developing after solid cancer (MN-SC). Methods: We retrospectively analyzed the clinicopathologic and genetic data of myeloid neoplasm patients diagnosed between 2008 and 2023, categorized as MN-BNHL or MN-SC. Further NGS analysis was performed on available bone marrow samples with missing genetic data. The genetic profiles of myeloid neoplasms between BNHL and solid cancer groups were compared. Results: Sixteen patients developed MN-BNHL. Among the 11 MN-BNHL patients undergoing NGS, all harbored tier 1 mutations. *PPM1D* mutations (PPM1Dms) were most frequent (73%), followed by *DNMT3A* (46%) and *TP53* (36%). PPM1Dms were significantly more prevalent than in MN-SC (*n* = 21), where *TP53* mutations were most common (64%) (*p* < 0.001). PPM1Dms often co-occurred with *DNMT3A*. They were associated with prior radioimmunotherapy (relative risk (RR): 3.3 and RR 3.57). MN-BNHL patients with PPM1Dms exhibited improved survival compared to those without (*p* = 0.0376), but this benefit was negated by the presence of *TP53* mutations (*p* = 0.0049). Conclusions: PPM1Dms are a prominent genetic feature in MN-BNHL, suggesting a distinct role in its development compared to MN-SC. Further investigation is needed to elucidate the precise contribution of *PPM1D* and its interaction with other mutations in BNHL-related myeloid neoplasm development and prognosis.

## Simple Summary

Myeloid neoplasms are a significant complication for B-cell non-Hodgkin lymphoma survivors treated with cytotoxic therapies. This study examined the genetic profiles of myeloid neoplasms following B-cell non-Hodgkin lymphoma, finding *PPM1D* mutations to be significantly more frequent than in myeloid neoplasms after solid cancers. *PPM1D* mutations, often with *DNMT3A*, were associated with improved survival unless *TP53* mutations were also present. This suggests the distinct role of *PPM1D* in myeloid neoplasms development after B-cell non-Hodgkin lymphoma.

## 1. Introduction

Non-Hodgkin lymphomas can be broadly classified as B-cell, T-cell, and NK-cell lymphomas, each with varying levels of aggressiveness and clinical presentations [1]. B-cell non-Hodgkin lymphoma (BNHL), a heterogeneous disease with overlapping histological and molecular subtypes, is the most common type of non-Hodgkin lymphoma [1]. While incidence varies globally, BNHL mortality has remained relatively stable or declined since 1997 [2]. BNHL accounts for 2.1% of all cancers, with about 5000 new cases per year [3]. Recently, the overall 5-year relative survival rate of non-Hodgkin lymphoma has improved to 74%, and it reached over 90% in follicular lymphoma [3]. Those achievements in NHL are attributed largely to advances in various treatments based on chemoimmunotherapy; the standard regimens to treat BNHL are cyclophosphamide, doxorubicin, vincristine (oncovin), and prednisone (CHOP), often combined with monoclonal antibody therapy such as rituximab. Although the prognosis is improving with the development of therapeutic agents, approximately 30–40% of patients with diffuse large B-cell lymphoma (DLBCL) experience relapse or refractory, especially within two years [4]. Additional types of therapy such as radiotherapy (RT) or radioimmunotherapy (RIT) are applied to those refractory or relapsed BNHL patients.

As the mortality of non-Hodgkin lymphoma has reduced and the average lifespan of populations has increased, the development of second primary cancer (SPC) after BNHL has increased [5]. Studies in Korea have reported a 5-year cumulative incidence of SPCs ranging from 7% to 15%, depending on BNHL subtype, over a 9-year follow-up [3]. While some studies suggest a higher risk of solid cancer compared to hematologic malignancies after BNHL, others have observed a greater prevalence of second primary hematologic cancers, particularly myeloid neoplasms (MNs) [6,7]. A recent large study conducted in the United States and Sweden confirmed that the risk of developing myelodysplastic neoplasms (MDS) or acute myeloid leukemia (AML) in BNHL survivors is more than five times higher than in the general population [5,7]. Risk factors for SPC development are diverse, with solid cancers potentially arising from direct DNA damage from treatments like alkylators, as well as hematopoietic stem cell transplantation [8].

The incidence of myeloid neoplasms post cytotoxic therapy (MN-pCT) is reported to be low (0.62 cases per 100,000) in all cancer survivors, but accounts for 10–20% of all MNs. Of these, 60% have a history of treatment for solid cancer and the remaining 40% are treated for hematological malignancies [9]. MN-pCT, encompassing MDS, AML, and myelodysplastic/myeloproliferative neoplasms, are distinct entities resulting from the DNA damage induced by prior cytotoxic therapies like chemotherapy and RT [10]. Specific therapy-related mutational patterns have been observed: alkylators are associated with *TP53* multi-hit lesions and longer latency (5–10 years), topoisomerase II inhibitors with balanced translocations involving *KMT2A* or *RUNX1* and shorter latency (1–3 years), and radiation with *EZH2* and *ETV6* mutations [5,11]. Recent CAR T-cell therapy, linked to post-therapy immunosuppression, exhibits a short latency to MNs (6–10 months) and is associated with *TP53* and *PPM1D* mutations in emerging clonal hematopoietic clones [9].

Myeloid neoplasms after BNHL (MN-BNHL) has been reported in several studies, with an estimated cumulative incidence of up to 10.5%, depending on the type and duration of the prior therapy, the subtype of B-cell lymphoma, and the length of follow-up [6,12]. MN-BNHL is usually preceded by MDS, which may remain undiagnosed until progression to AML. MN-BNHL has a dismal outcome, with a median OS of less than 12 months [13,14]. The poor prognosis of MN-BNHL is partly explained by the frequent occurrence of adverse cytogenetic and molecular abnormalities, such as complex karyotypes, monosomy 5 or 7, *TP53* mutation, and other mutations in genes involved in DNA methylation and splicing [15,16]. 

Emerging MN-BNHL represents a distinct subset of MN-pCT, with different clinical and biological features from myeloid neoplasms after solid cancer (MN-SC). However, most studies have focused on statistical data such as incidence and mortality on MN-pCT in BNHL [17]. The current knowledge on molecular pathogenesis and prognostic factors of MN-BNHL is limited. The mutational landscape and the clonal evolution of MN-BNHL are not fully understood and may differ from those of MN-SC. Therefore, there is a need for more comprehensive and comparative studies of MN-BNHL and MN-SC, using genomic data especially at the time of MN diagnosis. The aim of this study was to investigate the clinicopathological characteristics and mutational profile of MN-BNHL compared to MN-SC.

## 2. Materials and Methods

### 2.1. Patient

We conducted a retrospective cohort study of patients diagnosed with MN-pCT at the Korea Cancer Center Hospital between 2008 and 2023. We enrolled patients with a history of BNHL who subsequently developed and were diagnosed with MN-pCT. All patients had a history of exposure to cytotoxic therapy (chemotherapy, immunochemotherapy, RT, radioactive iodine (RAI) or RIT) for BNHL. We excluded patients based on the following criteria: (1) patients who had treated only surgery or non-cytotoxic therapies for the previous BNHL; and (2) patients with prior cytotoxic therapy for non-NHL cancers, to avoid confounding data on cytotoxic therapy-induced genetic alterations. Available bone marrow (BM) samples from patients with MN-BNHL were collected for genetic analysis of MN-associated genes.

### 2.2. Study Design

We divided patients into two groups based on first primary cancer (FPC): MN-BNHL and MN-SC. We compared the clinicopathologic and genetic data of these two groups. Clinical data on patient demographics, primary cancer subtype, cytotoxic treatment, MN subtype, survival status, and last follow-up date were collected from electronic medical records through an honest broker. To identify the genetic characteristics of MN-BNHL, we included patients with MN-SC who underwent genetic analysis with the same next-generation sequencing (NGS) panel at the time of their MN diagnosis. Genetic data from two MN-BNHL and all MN-SC patients were tested at the time of their MN diagnosis.

### 2.3. Samples Collection and Processing for NGS Analysis

The samples used in this study were obtained from the Korea Institute of Radiological and Medical Sciences (KIRAMS) radiation biobank (KRB) (KRB-2022-I005). Residual BM aspiration samples from the patients were collected in the form of buffy coat at the time of diagnosis of MN. The samples were stored in the refrigerator below −60 °C in the KRB. A total of nine BM samples were available and used for analyzing the genetic profile of MN using a NGS panel. Two samples from BNHL patients and all samples from solid cancer patients were tested at the time of diagnosis of MN. Genomic DNA was extracted from BM cells using a QIAamp DNA Blood Mini Kit (Qiagen, Venlo, The Netherlands) following the manufacturer’s protocol. 

### 2.4. Next-Generation Sequencing Analysis

Somatic gene mutation analysis with a NGS panel for 49 MN-associated genes was performed. A custom capture panel targeting coding exons and intron–exon boundaries of 49 genes related to MN was used (Table S1). Prepared libraries were hybridized with capture probes and sequenced as paired-end reads (2 × 150 bp) using MiSeq (Illumina, San Diego, CA, USA) with an average coverage of 700×. High-quality trimmed reads were aligned to the UCSC GRCh37 (hg19) reference genome with the Burrows–Wheeler aligner (BWA), version 0.7.17 [18]. Reads marked as PCR duplicates by Picard were excluded from further analysis (https://broadinstitute.github.io/picard/, accessed on 1 December 2022 and 2 September 2023). To ensure the best performance of MuTect2, the GATK BaseRecalibrator version 4.0.0.0 was used to increase the base quality score accuracy. Single-nucleotide variants and indels were called using Mutect2 and Varscan2 [19,20]. When there are overlapping calls for a variant between Mutect2 version and Varscan2, the Mutect2 results were prioritized and used. To detect FLT3-ITD, the Pindel algorithm was additionally used in analysis [21]. Mutation annotation was performed using the dbSNP, COSMIC, and ClinVar databases. All the genetic alterations were interpreted as a four-tier system according to standards and guidelines for the interpretation and reporting of sequence mutations in cancer: Tier I, mutations of strong clinical significance such as FDA-approved, professional guidelines or well-powered studies–appeared therapy; Tier II, mutations of potential clinical significance such as FDA-approved treatment for different tumor types or investigational therapies; Tier III, variants of unknown clinical significance; and Tier IV, benign of likely benign variants [22]. Variants corresponding to tier 4 (benign or likely benign) are not reported.

### 2.5. Statistical Analysis

Statistical analysis was performed using Rex version 3.6.3 (Rex Soft Inc., Seoul, Republic of Korea). Descriptive statistics, including mean, standard deviation (SD), median, interquartile range (IQR), frequency, and percentage, were calculated for clinicopathological and genetic characteristics. Differences in clinicopathological and genetic data between patients with MN-BNHL and MN-SC were assessed using the *t*-test and Fisher’s exact test or chi-square test, as appropriate. Continuous variables were compared between two independent groups using the Mann–Whitney U test or two-sample *t*-test, and among three or more independent groups using the Kruskal–Wallis test. Overall survival (OS) was estimated using the Kaplan–Meier method, with comparisons between groups performed using the log-rank test. Statistical significance was defined as *p* < 0.05. The genetic data were visualized using Oncoprinter and MutationMapper tools from cBioPortal for Cancer Genomics (https://www.cbioportal.org/visualize, accessed on 2 May 2024). The result in MutationMapper was modified with PowerPoint version 365 (Microsoft Co., Redmond, WA, USA).

## 3. Results

### 3.1. Characteristics of Patients with B-Cell Non-Hodgkin Lymphoma

Sixteen patients with MN-BNHL meeting the inclusion criteria were enrolled. The baseline patient characteristics are summarized in Table 1. The mean age at BNHL diagnosis was 56.7 years, and 63% of patients were male. Most BNHL patients presented at advanced stages, with 19% at stage III and 63% at stage IV. The most common primary BNHL subtype was DLBL (44%). Therapeutic strategies for the patients with BNHL varied between different subtypes and different conditions. All patients received chemotherapy (100%), rituximab-based immunochemotherapy (75%) and 44% and 31% received RIT and RT, respectively, for BNHL. No patient underwent autologous or allogeneic bone marrow transplantation. Moreover, 12 of 16 BNHL patients reached complete response or partial response a month after treatment, but 67% of the patients relapsed.

### 3.2. Genetic Features of Myeloid Neoplasms After B-Cell Non-Hodgkin Lymphoma

**Figure 1:**
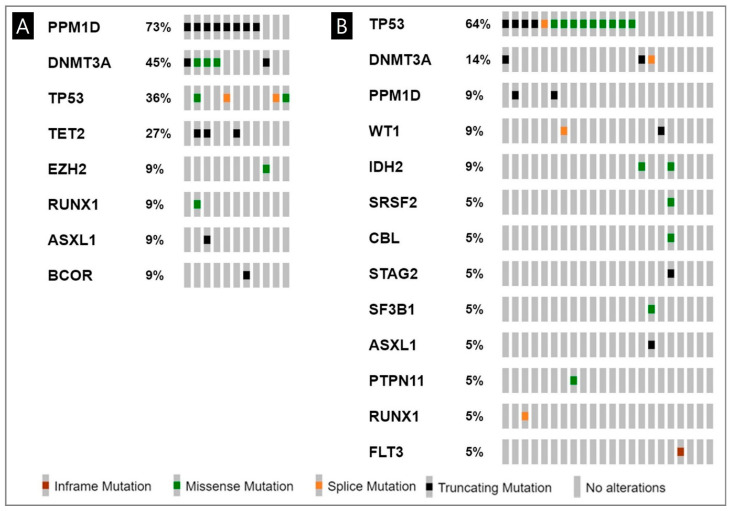
Tier 1 mutations in patients with myeloid neoplasms: (**A**) the frequency and type of mutated genes in patients with myeloid neoplasms following B-cell non-Hodgkin lymphoma; (**B**) solid cancers.

Of the 11 patients undergoing NGS analysis covering 49 MN-associated genes, all patients had more than one tier 1 mutation. All 39 mutations comprising tier 1, 2, and 3, with a mean of 4 mutations per patient (range: 1–6), are limited to 11 genes, and the tier 1 mutations are restricted to 8 genes (Figure 1A and Table S2). The most frequently mutated genes were *PPM1D* (73%), and *DNMT3A* (45%), *TP53* (36%), and *TET2* (27%) followed (Figure 1A).

**Figure 2:**
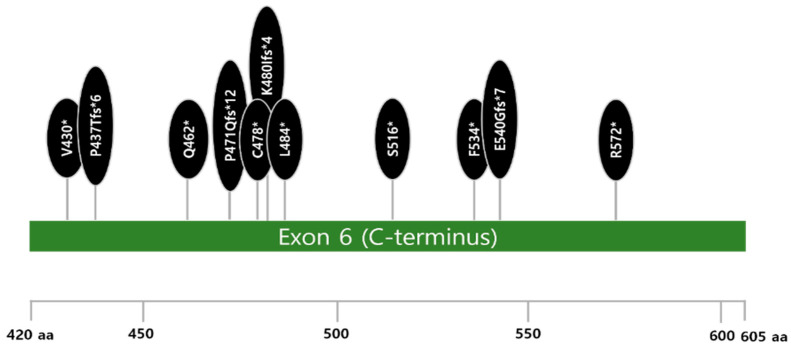
*PPM1D*-truncating mutations in patients with myeloid neoplasms following B-cell non-Hodgkin lymphoma. Abbreviation: aa, amino acid; * indicate the position of a stop codon in a new reading frame.

**Figure 3:**
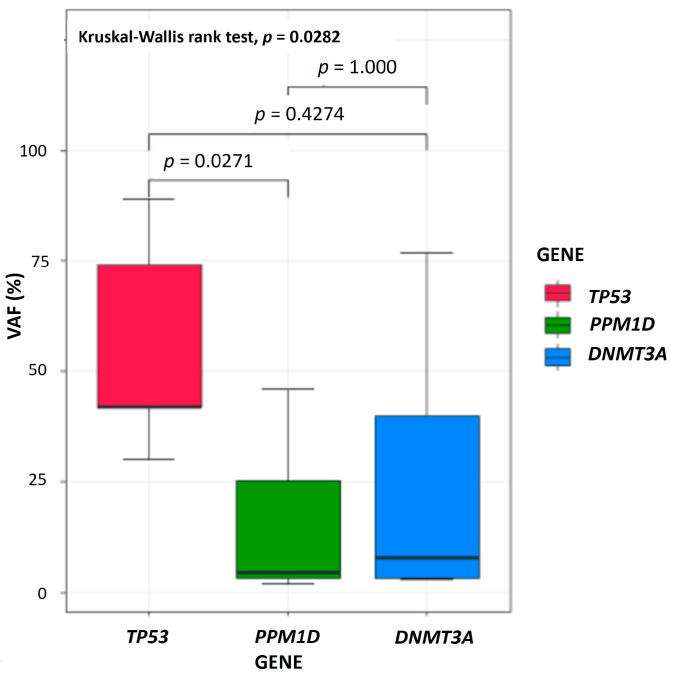
Variant allele frequency among three most frequently mutated genes in patients with myeloid neoplasms following B-cell non-Hodgkin lymphoma.

All PPM1Dms occurred in exon 6 with a type of truncation (100%) (Figure 2). They co-mutated with *DNMT3A* (50%), *TET2* (38%), and *TP53* (25%) mutations (Figure 1A and Table S1). Two of eight patients with PPM1Dms had more than 2 or 3 PPM1Dms in different alleles. A significant difference in variant allele frequency (VAF) (%) among the three most frequently mutated genes was seen (*PPM1D*, *TP53*, *DNMT3A*) (*p* = 0.0282) and the VAF (%) of PPM1Dms was much lower than that of *TP53* mutations (TP53ms) (*p* = 0.0271) (Figure 3).

### 3.3. Comparison of Clinicopathologic and Genetic Features of Myeloid Neoplasms After B-Cell Non-Hodgkin Lymphoma and Solid Cancer

Twenty-one MN-SC patients with clinicopathologic and genetic data were included for comparison with data from MN-BNHL patients (Table 2 and Table S3). In treatment exposures, rituximab and internal radiation therapy including RIT or RAI was more common in MN-BNHL than in MN-SC (both, *p* < 0.001). Longer interval periods to develop MN were observed after diagnosis of first primary solid cancer than after BNHL (*p* = 0.0305). Dysmegakaryopoiesis and BM fibrosis were more pronounced in the BM of MN-SC rather than in that of MN-BNHL (*p* = 0.0265 and *p* < 0.001, respectively).

We compared the mutational profile of MN-BNHL (*n* = 11) with that of MN-SC (*n* = 21) using the same targeted NGS of 49 genes related to MN pathogenesis. The most frequently mutated tier 1 genes in each group were PPM1Dms (73%) and TP53ms (64%), respectively (Figure 1A,B). *PPM1D* and *DNMT3A* mutations occurred more commonly in MN-BNHL than in MN-SC (*p* < 0.001 and *p* = 0.0318, respectively). Relative risks (RR) of mutations in three genes with high frequency (*PPM1D*, *TP53*, and *DNMT3A*) were shown with exposure to a certain type of cytotoxic therapy (Table 3). Exposure to rituximab and RIT/RAI was associated with increased risks of PPM1Dms (RR = 3.67, 95% CI: 1.08–12.43; RR = 3.3, 95% CI: 1.19–9.16). Similarly, RIT/RAI exposure was associated with an elevated risk of *DNMT3A* mutations (RR = 3.57, 95% CI: 1.43–8.90).

### 3.4. Survival Analysis

**Figure 4:**
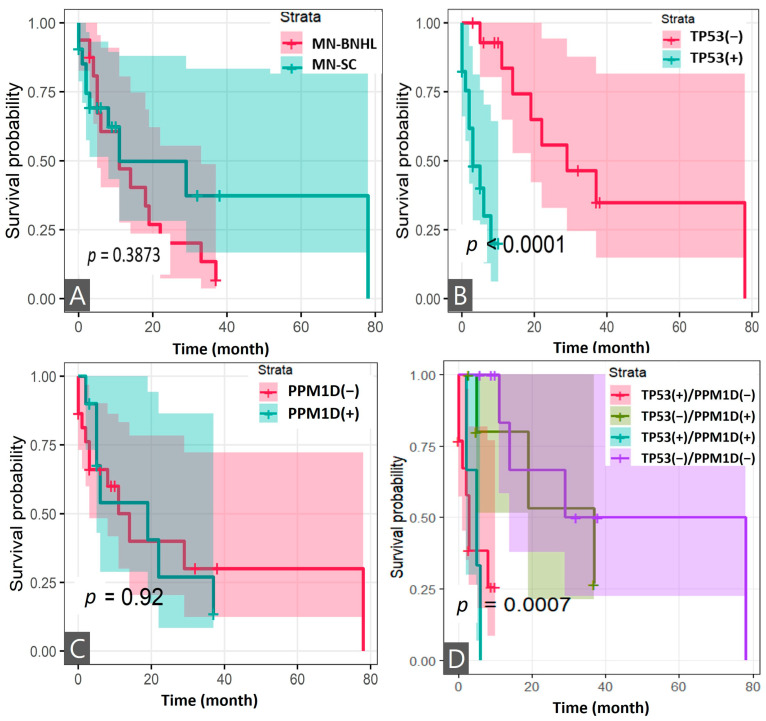
Survival analysis of patients after diagnosis of myeloid neoplasms post cytotoxic therapy: (**A**) overall survival in patients with myeloid neoplasms post cytotoxic therapy following B-cell non-Hodgkin lymphoma and solid cancers (*n* = 37); (**B**) survival analysis by *TP53* mutation status (*n* = 32); (**C**) survival analysis by *PPM1D* mutation status (*n* = 32); and (**D**) survival analysis by *TP53* and *PPM1D* mutation status (*n* = 32).

**Figure 5:**
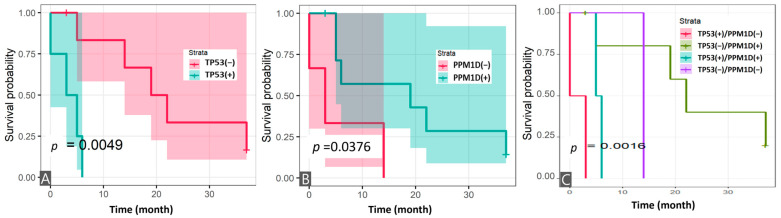
Survival analysis of patients with myeloid neoplasms post cytotoxic therapy following B-cell non-Hodgkin lymphoma (*n* = 11): (**A**) survival analysis by *TP53* mutation status; (**B**) survival analysis by *PPM1D* mutation status; and (**C**) survival analysis by *TP53* and *PPM1D* mutation status.

The mean follow-up time for all patients after FPC was 10.9 years (range: 1.3–28.4). Patients with MN-BNHL showed a median OS of 11 months (95% CI: 4–22) after MN diagnosis, compared to 6 months (95% CI: 2–10) for MN-SC patients (*p* = 0.1574) (Table 2). Kaplan–Meier analysis revealed no significant difference in OS between the two groups (log-rank, *p* = 0.3873) (Figure 4A). Analysis of *TP53* and *PPM1D* mutations in 32 MN patients revealed that patients with TP53ms demonstrated significantly shorter survival compared to those without (log-rank, *p* < 0.0001), while PPM1Dm status alone did not significantly impact survival (Figure 4B,C). Further stratification into four groups based on mutation status combinations (TP53m+/PPM1Dm−; TP53m−/PPM1Dm+; TP53m+/PPM1Dm+; and TP53m−/PPM1Dm−) showed significant differences in survival outcomes (log-rank, *p* = 0.0007) (Figure 4D). In the MN-BNHL analysis, patients harboring TP53ms demonstrated significantly shorter survival (log-rank, *p* = 0.0049), while those with PPM1Dm showed improved survival (log-rank, *p* = 0.0376) (Figure 5A,B). Analysis of the four mutation status combinations with *TP53* and *PPM1D* genes in this subgroup also revealed significant survival differences (log-rank, *p* = 0.0016) (Figure 5C).

The 95% confidence intervals (shadows for each survival curve) are displayed for all survival curves.

The 95% confidence intervals are displayed for all survival curves except Figure 4C. Due to the presence of zero values within the standard error calculations for this specific group, the confidence intervals could not be reliably determined.

## 4. Discussion

Currently, there are limited studies elucidating the genetic alterations associated with the development of MNs in patients with BNHL, despite the recognized increased risk of MNs in BNHL survivors [9,23]. MN-pCT, a distinct category of hematologic cancers, arises from clonal hematopoiesis, a condition where mutated pre-cancerous cells expand [10]. Cytotoxic (DNA-damaging) therapy in an altered BM environment can further select and expand these pre-existing clones, contributing to MN-pCT development [1]. While most studies on MN-pCT have highlighted a strong association with TP53ms, our study identified *PPM1D* as the most frequently mutated gene in MN-pCT following BNHL, particularly when compared to MN-pCT arising after solid cancers. The observation of PPM1Dms as a prominent feature in MN-BNHL in our cohort aligns with the understanding that prior cytotoxic exposure can drive the development of genetically distinct MNs. These data support the concept that treatment-related exposures greatly impact the genetic make-up of these secondary malignancies. The discrepancy in the most frequently mutated gene, when compared to MN-pCT arising after BNHL and solid tumors, emphasizes the complex interplay of treatment-related factors, individual patient characteristics, and the underlying biology of MN-pCT. The association of PPM1Dms with rituximab-based immunochemotherapy and RIT in this study necessitates further investigation into its role and potential therapeutic targets.

*PPM1D*, a serine/threonine phosphatase, plays a crucial role in regulating cell growth and proliferation and interacts with proteins involved in cell cycle control and DNA damage response, including p53, c-Myc, and MDM2 [24]. PPM1Dms, prevalent in 20% of diffuse large B-cell lymphoma, are associated with inferior outcomes and CAR T-cell therapy resistance [25]. In addition, PPM1Dms harbored approximately 20% of MN-pCT in patients [26]. Studies have suggested that DNA damage response genes, such as *TP53*, *PPM1D*, and *CHCK2*, can serve as biomarkers of prior genotoxic stress, particularly platinum exposure, and are associated with MN-pCT risk [27,28]. PPM1Dms are thought to contribute to MN-pCT development by inactivating p53, thereby allowing cells to accumulate further mutations, although the precise mechanisms are still under investigation. The selective outgrowth of *PPM1D*-mutant hematopoietic cells following chemotherapy exposure, both in vitro and in vivo, can occur through truncating PPM1Dms, particularly in exon 6. This leads to decreased protein degradation due to the loss of a C-terminal degradation signal and the subsequent overexpression of the PPM1D protein [29]. PPM1D overexpression has also been observed in both solid cancers and MN-pCT, and can have similar effects to PPM1Dms [30].

Previous studies have indicated that MN-pCT patients with *PPM1D* mutation or overexpression have a worse prognosis [30]. Consistent with previous findings, all PPM1Dms observed were truncating mutations located in exon 6, with relatively low VAFs (median 4.6%, IQR: 3.4–25.3%) compared to the VAFs of TP53ms in our study (Figure 3) [24,30,31]. Functional studies have suggested that *PPM1D* truncating mutations promote MN-pCT development by disrupting normal hematopoiesis, allowing cells to escape chemotherapy-induced cell death [31]. A recent report indicated that the frequency of somatic mutations in cancer is associated with overall gene expression, with premature terminating mutations in *TP53*, *ARID1*, and *NSD1*, particularly those with high allele frequencies, playing a significant role in cancer development [32]. However, studies in CLL have shown that TP53ms are associated with poor prognosis regardless of VAF [33]. While recent reports suggest that pre-existing genomic mutations may not directly predict MN-pCT development following cytotoxic therapy, the increased risk of MNs may arise from subclones within the hematopoietic cell lines that acquire additional mutations during disease progression and treatment [28,34,35,36,37]. Moreover, a previous study identified shared mutations between lymphoma and MN in some patients, with a germline mutation observed in one patient, suggesting a potential role for genetic predisposition alongside acquired mutations during lymphoma development [38].

The prognosis of patients with MN-pCT was generally poor, with an overall 5-year survival rate of 10% [5]. Prognosis is significantly influenced by associated genetic profiles and comorbidities. Consistent with previous studies, TP53m was a strong indicator of poor prognosis in both MN-BNHL and total MN patients in our study. Interestingly, we observed a better prognosis in patients with PPM1Dms compared to those with TP53ms (Figure 4). The precise pathogenic role of PPM1Dms in MN-BNHL remains unclear, but it is possible that they cooperate with other mutations, such as *TP53* or *DNMT3A*, to impair DNA damage response and cell cycle control, leading to genomic instability and malignant transformation. A recent report presented the synergistic effect of inhibiting both MDM2 and PPM1D, showing that dual inhibition leads to a more pronounced apoptotic response in cancer cells [39]. In murine models, elevated PPM1D expression results in tumors with phenotypic characteristics similar to those observed with *TP53* loss-of-function mutations [40]. This is attributed to *PPM1D*’s function as a negative regulator of the p53 protein, whereby its overexpression leads to *TP53* inhibition and the promotion of tumor development [26,40]. However, only three patients in our MN-BNHL group were *PPM1D*-negative, and two of them harbored TP53ms. Despite of the limited number of cases, the contrasting mechanisms of these mutations—TP53ms resulting in loss of tumor suppression and genomic instability, and PPM1Dms partially suppressing p53 while maintaining some DNA damage response function, allowing survival under genotoxic stress with a stable, low clonal burden—likely contribute to the observed clinical outcomes.

This study has several limitations. The retrospective design inherently carries the risk of selection bias and limits the availability of comprehensive clinical data and BM samples. Specifically, detailed information regarding specific therapeutic regimens, including the duration and intensity of cytotoxic therapies, was not consistently available, limiting our ability to fully assess the impact of these treatments on MN-pCT development and the emergence of specific genetic alterations, including germline predispositions. The relatively small sample size, while representative of a rare disease manifestation, limits the statistical power of our analyses and may affect the generalizability of our findings. Larger, multi-center studies are needed to validate our observations and further explore the clinical significance of PPM1Dms in MN-BNHL. The absence of serial BM samples prevented us from tracking the genetic evolution from BNHL to MN, hindering our ability to determine the precise timing of *PPM1D* mutation acquisition and characterize clonal dynamics. Future studies incorporating longitudinal sampling are essential. Furthermore, we acknowledge the potential role of the clonal hematopoiesis of indeterminate potential (CHIP) in MN-pCT development. The absence of pre-diagnostic genetic profiling limited our ability to distinguish between mutations acquired during CHIP and those arising de novo during BNHL or MN-pCT development. Future studies should investigate CHIP prevalence in BNHL patients and its association with MN-pCT risk. The heterogeneity of our MN-SC comparison group, encompassing various cancer types, may have introduced confounding factors and limited the specificity of our comparative analysis. The findings and their implications should be discussed in the broadest context possible. Future research directions may also be highlighted.

## 5. Conclusions

In summary, our study highlights *PPM1D* as a prominent genetic alteration in MN-BNHL, distinguishing it from MN-SC, where TP53ms are more prevalent. This finding suggests a unique pathogenic mechanism in MN arising after BNHL. The potential of *PPM1D* as a therapeutic and monitoring target for both BNHL and MN-pCT warrants further investigation. Future studies should focus on validating these findings in larger, prospective cohorts and exploring the functional consequences of PPM1Dms in MN-pCT pathogenesis.

## Figures and Tables

**Table 1:** Characteristics with B-cell non-Hodgkin lymphoma (*n* = 16).

Characteristics	BNHL (*n* = 16)
Age of diagnosis of BNHL, years	56.7 ± 11.5
Male gender, n (%)	10 (63%)
Stage *	
I	1 (6%)
II	2 (13%)
III	3 (19%)
IV	10 (63%)
Pathologic diagnosis	
DLBL	7 (44%)
FL	4 (25%)
MZBL CLL MCL	2 (13%) 1 (6%) 2 (13%)
Cytotoxic therapy exposure	16 (100%)
Chemotherapy only	2 (13%)
Chemotherapy + RIT	1 (6%)
Chemotherapy + RIT + RT	1 (6%)
Immunochemotherapy (rituximab) combination	12 (75%)
Immunochemotherapy	2 (13%)
Immunochemotherapy + RIT	7 (44%)
Immunochemotherapy + RIT + RT	3 (19%)
Treatment response (IWC + PET criteria)	
CR	10 (63%)
PR	2 (13%)
SD	2 (13%)
PD	1 (6%)
NA	1 (6%)
Relapse	
NA	1 (8%)
CR or PR	3 (25%)
Relapse	8 (67%)

Abbreviation: BNHL, B-cell non-Hodgkin lymphoma; CLL, chronic lymphocytic leukemia; CR, complete response; DLBL, diffuse large B-cell lymphoma; FL, follicular lymphoma; IWC, international workshop criteria; MCL, mantle cell lymphoma; MZBL, marginal zone B-cell lymphoma; NA, not available; PD, progressive disease; PET, positron emission tomography; PR, partial response; RIT, radioimmunotherapy; RT, radiotherapy; and SD, stable disease. Data are represented as mean ± SD for normally distributed continuous variables, and n (%) for categorical variables. Radioimmunotherapy involved Iodine-131 in 11 patients (69%) and Yttrium-90 in 1 patient (6%). * Stages were based on Lugano staging classification.

**Table 2:** Comparisons of clinicopathologic and genetic characteristics between myeloid neoplasms after B-cell non-Hodgkin lymphoma and solid cancers.

Characteristics	MN-BNHL (*n* = 16)	MN-SC ^†^ (*n* = 21)	*p*-Value
Age, years at diagnosis of MN	66.5 (57.5, 72)	63 (57, 67)	0.3735
Exposure to cytotoxic therapies			
Chemotherapy	16 (100%)	19 (90%)	0.4955
Rituximab	12 (75%)	0 (0%)	<0.001
Radiotherapy	4 (25%)	12 (57%)	0.1052
RIT/RAI	12 (75%)	1 (5%)	<0.001
Interval period, year (range)	7.0 ± 4.0 (2.4 to 17.3)	11.7 ± 8.3 (1.3 to 28)	0.0305
Survival state			
Death	14 (88%)	10 (48%)	0.03
Survival period after MN, month (range)	11 (4.8–19.8)	6 (2–10)	0.1574
Follow-up period, year (range)	9.0 ± 4.3 (2.8–18.6)	12.4 ± 8.4 (1.3–28.4)	0.1183
BM study			
Blast count (%)	6.9 (4.5, 16.9)	14.6 (6, 46.6)	0.2696
Dysplasia (>10%)			
Dyserythropoiesis	10 (63%)	8 (42%)	0.388
Dysgranulopoiesis	2 (13%)	4 (21%)	0.6657
Dysmegakaryopoiesis	6 (43%)	15 (83%)	0.0265
BM Fibrosis (≥MF − 1)	0 (0%)	10 (59%)	<0.001
Chromosomal aberrations			
Complex karyotype	11 (69%)	13 (62%)	0.9326
–7/del7q	10 (63%)	8 (38%)	0.2545
–5/del5q	4 (25%)	9 (45%)	0.3722
i (17q), –17/add(17p) or del(17p)	4 (25%)	1 (5%)	0.1444
–20/del20q	1 (6%)	3 (15%)	0.6129
Somatic mutations associated with myeloid neoplasms * (3 dominant tier-1-mutated genes)			
*PPM1D*	8 (73%)	2 (10%)	<0.001
*TP53*	4 (36%)	13 (62%)	0.2818
*DNMT3A*	5 (45%)	2 (10%)	0.0318

Abbreviations: BM, bone marrow; MN-BNHL, myeloid neoplasms after b-cell non-Hodgkin lymphoma; MN-SC, myeloid neoplasms after solid cancer; RIT, radioimmunotherapy; and RAI, radioactive iodine. Data are represented as mean ± SD for normally distributed continuous variables, median (interquartile range) for non-normally distributed continuous variables, and n (%) for categorical variables. Radioimmunotherapy involved iodine-131 in 11 BNHL patients and Yttrium-90 in 1 BNHL patient, and radioiodine therapy was to a thyroid cancer patient. ^†^ Includes breast cancer (*n* = 7), ovarian cancer (*n* = 3), breast and ovarian cancer (*n* = 2), gastric cancer (*n* = 2), sarcomas (*n* = 2), laryngeal cancer (*n* = 1), lung cancer (*n* = 1), rectal cancer (*n* = 1), thyroid cancer (*n* = 1), and astrocytoma (*n* = 1). * Genetic data from 11 MN-BNHL and 21 MN-SC patients were analyzed.

**Table 3:** Mutation risks (*PPM1D*, *TP53*, and *DNMT3A*) by cytotoxic therapy type in myeloid neoplasms patients (*n* = 32).

Treatment/Gene Mutation	Relative Risk (95% CI)			
	Chemotherapy	Rituximab (Immunochemotherapy)	RIT/RAI	Radiotherapy
PPM1Dm (+)	1.07 (0.88, 1.30)	3.67 (1.08, 12.43)	3.3 (1.19, 9.16)	0.51 (0.19, 1.39)
TP53m (+)	1.15 (0.92, 1.44)	0.88 (0.26, 2.92)	0.59 (0.20, 1.69)	1.13 (0.56, 2.29)
DNMT3Am (+)	1.03 (0.83, 1.29)	2.14 (0.67, 6.83)	3.57 (1.43, 8.90)	0.51 (0.15, 1.73)

Abbreviations: CI, confidence interval; RIT, radioimmunotherapy; and RAI, radioactive iodine.

## Data Availability

The data presented in this study are available upon request from the corresponding author.

## Supplementary Materials

The following supporting information can be downloaded at: https://www.mdpi.com/article/10.3390/cancers17071185/s1, Table S1: List of 49 genes and target regions in the myeloid neoplasm NGS panel; Table S2: Gene profile of patients with myeloid neoplasms post cytotoxic therapy (*n* = 32); Table S3. Clincopathological and genetic data of total patients (*n* = 37).
